# First African thylacocephalans from the Famennian of Morocco and their role in Late Devonian food webs

**DOI:** 10.1038/s41598-020-61770-0

**Published:** 2020-03-20

**Authors:** Melina Jobbins, Carolin Haug, Christian Klug

**Affiliations:** 1 University of Zürich, Zürich, Switzerland; 2 University of Munich, Munich, Germany

**Keywords:** Palaeontology, Evolution, Ecology

## Abstract

Thylacocephalans are enigmatic arthropods with an erratic Palaeozoic and Mesozoic fossil record. In many of the few localities where they occur, they are quite abundant. This also holds true for the Famennian Thylacocephalan Layer in the Maider (eastern Anti-Atlas of Morocco), a small epicontinental basin hosting some strata with taphonomic properties of a conservation deposit yielding exceptionally preserved gnathostomes and non-vertebrates. In a thin argillaceous interval in the earliest middle Famennian, thylacocephalans occur in such great numbers that they became eponyms of this unit. Therein, we discovered a new taxon of thylacocephalans, *Concavicaris submarinus* sp. nov., which represent the oldest records of thylacocephalans from Africa. In the CT-imagery, the holotype of *Concavicaris submarinus* sp. nov. revealed anatomical details including its eyes, appendages and other soft parts. Sedimentary facies and faunal composition of the Thylacocephalan Layer suggest that these animals populated the water column above the low-oxygen sea floor. Thus, thylacocephalans likely represented an important component of the diet of chondrichthyans and placoderms, which are quite common as well. The abundance of thylacocephalans in other conservation deposits like the Cleveland Shale (USA) and the Gogo Formation (Australia) underline their pivotal role in Late Devonian pelagic food webs.

## Introduction

Thylacocephalans constitute a group of small to medium-sized marine arthropods of unclear affinities. Their first undisputed appearance in the fossil record dates back to the Silurian^1^, although some fossils of Early Cambrian age^2^ may also belong to this group. Thylacocephalans lived until the Late Cretaceous period; the youngest fossils were found in the Cenomanian of Lebanon^3–5^.

Their key anatomical features include a body almost entirely enclosed within a bivalved carapace (in the wide sense) that usually carries a pointed rostrum in the front. Below the rostrum, a pair of multi-facetted compound eyes was situated. These eyes reached giant proportions in Mesozoic forms^1,6^. Three pairs of subchelate raptorial appendages are located at the front of the body, but it is not entirely clear from which segments they arise^1^. Eight pairs of lamellate gills and a series of at least eight pairs of small paddle-like limbs were present at the posterior part of the body^1^. Although various anatomical features are known from exceptionally well-preserved specimens, many questions related to their palaeobiology and systematic position are yet to be resolved^6^. For example, their systematic position remains disputed due to unknown anatomical features such as their cephalic appendages. A recent finding from the Silurian of the US suggested the presence of antennae in a very-well preserved specimen, which would increase the likelihood that they really belong to the crustaceans^1^, but this character may not be very reliable^1^. Additionally, Haug *et al*. points out that the presence of several enditic structures on the legs supports a position in the Eucrustacea^1^. Thylacocephalans from the Devonian are known to occur in a few places in Europe^5,7–9^, Australia^10^ and North America (e.g.)^11^. The Moroccan occurrence described here represents, as far as we know, the first record of thylacocephalans from Africa. This is also the second report of African thylacocephalans., the other being from the Early Triassic of Madagascar^12^.

Late Devonian strata of the eastern Anti-Atlas are known to be highly fossiliferous, yielding both vertebrates and invertebrates^13–16^. The Middle Famennian outcrops contain a layer attributed to the *Maeneceras* genozone^13,17–19^, erroneously called ‘Phyllocarid Layer’ in an earlier paper (Ref.^15^, renamed in^20^). The specimens presented here were found along with other invertebrates such as cephalopods and vertebrates including actinopterygians, chondrichthyans, placoderms and sarcopterygians. The Thylacocephalan Layer crops out at many localities in the southern Maïder such as Bid er Ras, Jebel Oufatene, Mousgar, Tizi n’Aarrat Chouiref, Oued Chouiref, Tizi Mousgar, Aguelmous Azizaou and Madène El Mrakib^15^. However, this layer is best accessible between Tafraoute, Madène El Mrakib and Mousgar, thus having produced the majority of our specimens.

Within this study, we present a new thylacocephalan species from this region. We also describe an exceptionally well-preserved specimen of *Concavicaris submarinus* sp. nov. that sheds new light on anatomical features that have not been documented before from thylacocephalans. Also, the ecological properties of these animals are discussed in their synecological context in order to reconstruct properties of their habitat and their position in the food web.

## Results

### Systematics

**Thylacocephala** *sensu* ^21^

Concavicarida *sensu*^10^

***Concavicaris**** sensu*^22^.

### Diagnosis

“Optic notches generally modest in size, prominently defined, dorsally delineated by a variously developed rostrum, ventrally by variously developed anteroventral processes. Posterior aspect of carapace truncated but gently rounded” (Schram, 2014^23^).

### Remarks

*C. submarinus* is assigned to the genus *Concavicaris* based on the following morphological characters: optic notch modest in size and the posterior of the carapace is truncated and gently rounded. Additionally, the rostum and anteroventral process are variously developed among the genus, which does not disagree with the rostrum and anteroventral process observed in *C. submarinus*.

Species *Concavicaris submarinus* sp. nov.

### Etymology

From the Latin word *submarinus* referring to submarine, as their shape is reminiscent of these watercrafts.

### Holotype

PIMUZ 37349, which is an exceptional specimen preserving both eyes, posterior limbs and some internal structures (visible in the CT-images).

### Paratypes

PIMUZ 37348, PIMUZ 37350, PIMUZ 37351, PIMUZ 37353, PIMUZ37354, AA.MEM.DS.3, AA.MEM.DS.4 and AA.MEM.DS.5.

### Nomenclatural statement

A Life Science Identifier (LSID) was obtained for the new species (*C. submarinus*): urn:lsid:zoobank.org:act:XX, and for this publication: urn:lsid:zoobank.org:pub:XX.

### Material

Although there are many fossils in the field and some tens of specimens were collected, we selected the best preserved specimens for the description (PIMUZ 37348–51, PIMUZ 37353–4 and AA.MEM.DS.3, AA.MEM.DS.4 and AA.MEM.DS.5). The size of the specimens varies from 27 mm (PIMUZ 37354) to 98 mm (PIMUZ 37348) in length and 10 mm (PIMUZ 37354) to 46 mm (PIMUZ 37348) in height.

### Locality and horizon

Madène El Mrakib, Morocco; *Maeneceras* genozone, middle Famennian, Late Devonian.

### Diagnosis

The optic notch has a small anteroventral process. The mid-ventral margin of the carapace curves towards the outside. A depression is visible along the curved margin, beginning shortly after the curve anteriorly and forming a longitudinal line until it reaches about 2/3 of the carapace length. There is no sign of ornamentation except the polygonal pattern of the cuticle.

### Description

**Figure 1:**
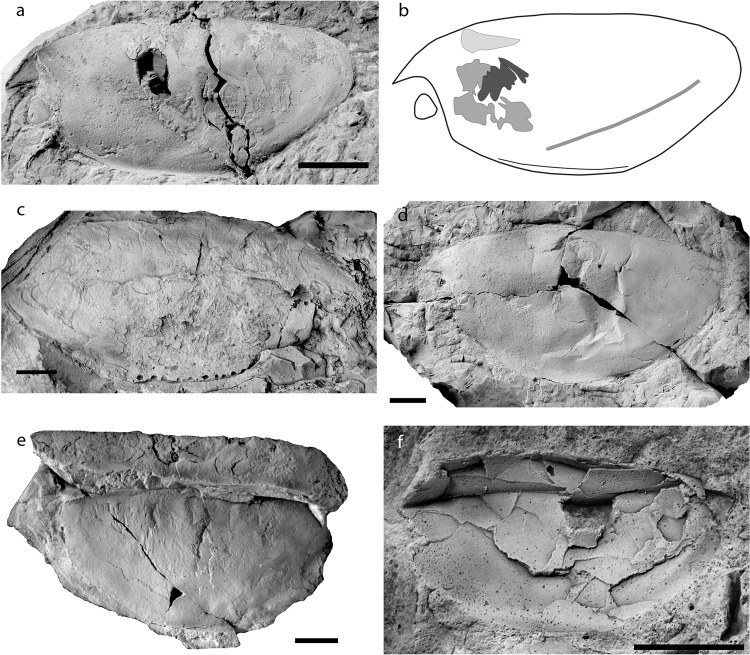
Specimens of *Concavicaris submarinus*. (**a**) Holotype PIMUZ 37349. (**b**) Line drawing of the holotype, showing the location of the ventral curve, the depression lying from anterior the rear end until midway through the carapace, and the internal structures (section of the gills in black, putative gastric muscles in dark grey and paired structure in light grey). (**c**) PIMUZ 37348, the biggest specimen, lacks the rear end. (**d**) PIMUZ 37350. (**e**) PIMUZ 37353. (**f**) PIMUZ 37354, the smallest specimen. Anterior is on the left for (**a**,**b**,**d**,**f**). Anterior is on the right for (**c**,**e**). Scale bars indicate 10 mm.

The holotype, PIMUZ 37349, is 56 mm long, 26 mm high and 15 mm wide (Fig. 1a). The biggest specimen available to us, PIMUZ 37348, lacks the posterior end, but its total length can be estimated to have reached nearly 100 mm at a height of 46 mm (Fig. 1c). PIMUZ 37354 lacks the rostrum and is the smallest specimen, which is 27 mm long and 10 mm high (Fig. 1f). AA.MEM.DS.4 and PIMUZ 37354 lack their rostrum. All specimens reach the maximum carapace height anterior to the centre.

There is no posterior spine in any of the specimens. When the rear end is preserved, it appears always truncated and gently rounded. The anteroventral process of the optic notch is small in size and visible in most specimens (Fig. 1).

**Figure 2:**
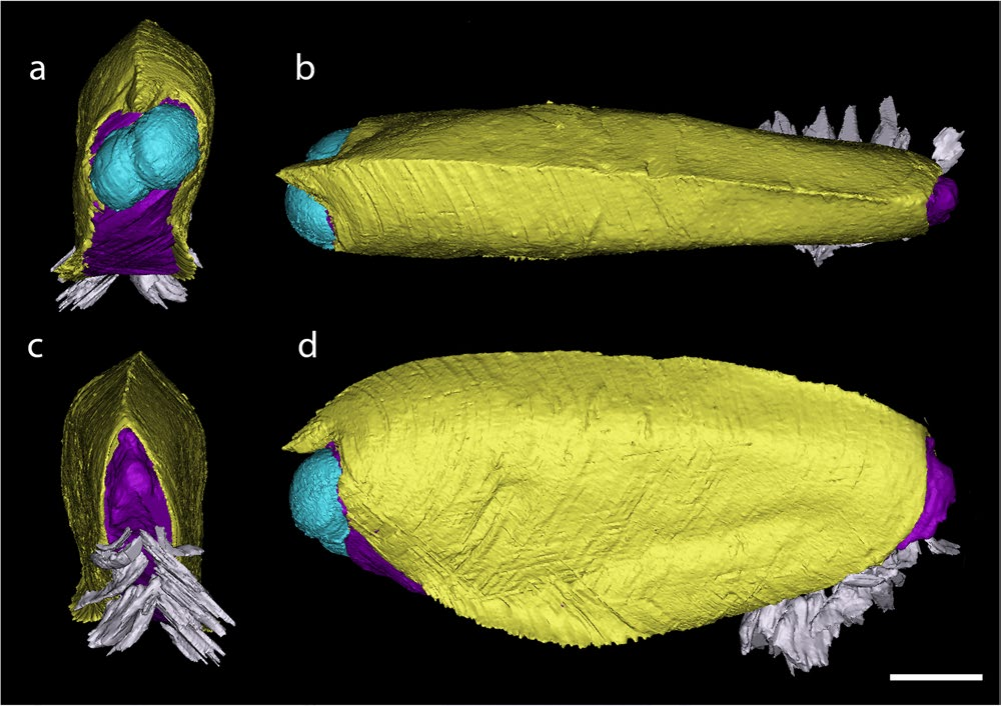
3D reconstruction of PIMUZ 37349, the holotype of *Concavicaris submarinus*. (**a**) frontal view. (**b**) posterior view. (**c**) dorsal view. (**d**) lateral view. The carapace is here in yellow, the main body appears in purple, the eyes are in blue and the paddle-like limbs in white. Scale bar indicates 10 mm.

The most noticeable diagnostic feature of *Concavicaris submarinus* lies on the ventral margin (Figs. 1 and 2). This edge has a section anterior to the centre, which is bent outwards, i.e. laterally (e.g. Fig. 1a,d,e). This is best visible in the least deformed specimens (AA.MEM.DS.3 and 4, PIMUZ 37348, 37349, 37353, 37354). Along the ventral margin, a depression runs longitudinally from the anterior starting point of the bent margin along a third of the carapace length (Fig. 1a,b).

**Figure 3:**
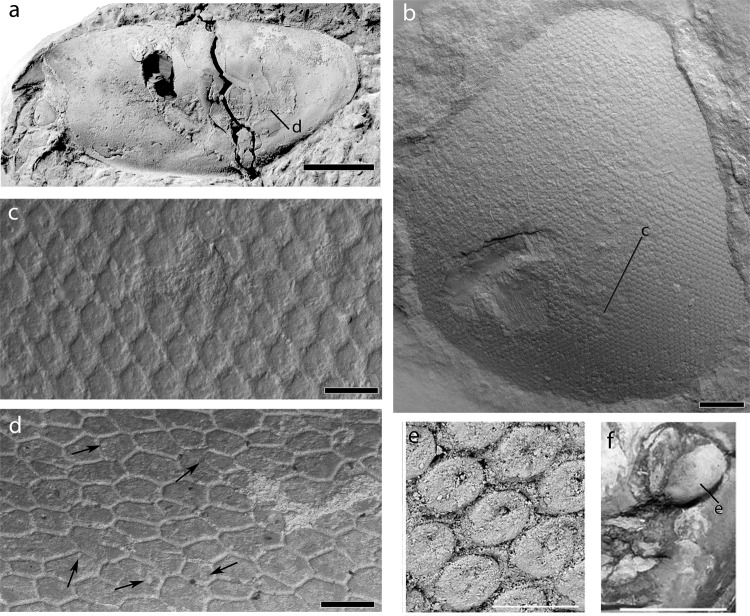
SEM images of the eye surface structure and cuticle of the carapace of the holotype of *Concavicaris submarinus* n. sp. (PIMUZ 37349; **a**–**d**) (**a**) the entire specimen, coated with NH_4_Cl-sublimate. (**b**) detail showing of the eye. (**c**) close-up of the hexagonal facets of the eye. (**d**) a section of the cuticle, visible on most of the carapace, made of polygons, some of which show a round depression (indicated by black arrows). (**e**) close up of the hexagonal facets of the eye of *Dollocaris*. (**f**) detail showing of the eye of *Dollocaris* and place where (**e**) was made. (**e**,**f**) were modified from^26^ Scale bars are (**b**) 500 µm, (**c**) 100 µm, (**d**) 200 µm, 50 µm (**e**) and 10 mm (**f**).

The large compound eyes of *Concavicaris submarinus* are well preserved in the holotype and reach a seventh of the body length (see on Fig. 2). Their structure is clearly visible under the microscope and shows a clear and regular pattern of convex, slightly elongated, hexagonal facets (Fig. 3a–c).

The carapaces of eight specimens preserve the cuticle in several places. Its good preservation in various areas of the carapace (e.g., PIMUZ 37349) reveals the polygonal imprints of cuticle cells, bordered by thin grooves (Fig. 3d). The polygons are irregular in the number of sides, overall shape and size. Some cells also show a more or less central depression (see black arrows on Fig. 3d).

**Figure 4:**
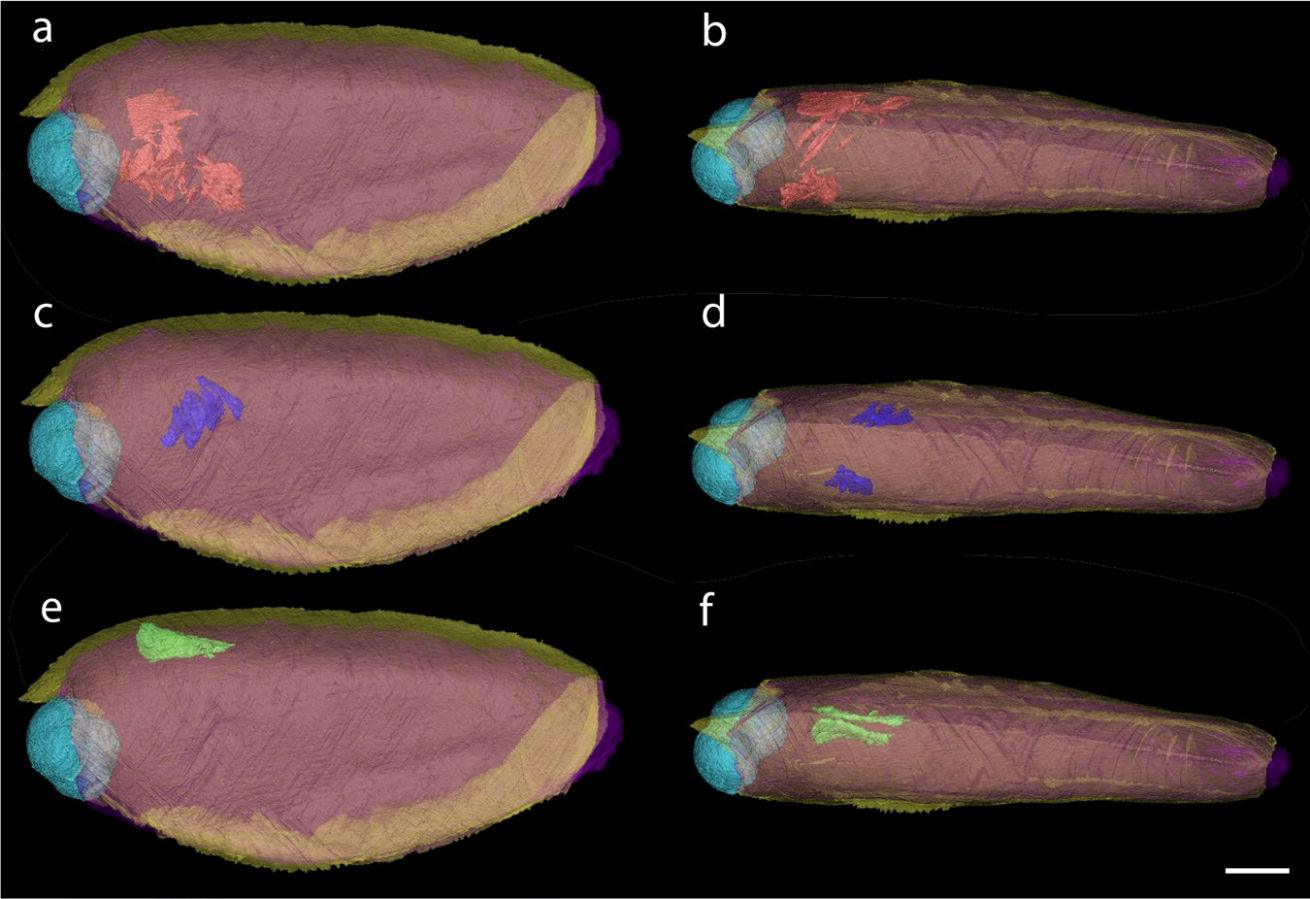
Internal structures of *Concavicaris submarinus* (PIMUZ 37349). The presumed gastric muscles appear here in red and are seen in a lateral (**a**) and dorsal (**b**) view. The sections of the putative gills are shown in blue, in both lateral (**c**) and dorsal (**d**) view. The elongated structures, interpreted as gonads or the hepatopancreas are shown in green and in a lateral (**e**) and dorsal (**f**) view. Scale bar is 10 mm.

In PIMUZ 37349 eight pairs of paddle-like limbs are preserved at the posteroventral end (only visible in the CT-images) – one leg per side being incomplete (Fig. 2). The legs on its left side are taphonomically deformed, making them appear much thicker (6.1 mm compared to 2.4 mm on the other side) than long (6.5 instead of 14 mm on the other side). Because of the deformation, it is conceivable that the legs have been pushed outwards, which could explain why many of them seem to be detached from the body. The legs expand from posterior of the curved ventral margin until shortly posterior of the carapace where they reach out of the carapace. The trunk segments are not visible in the scan (Fig. 4), either because they did not preserve or because of an insufficient density contrast.

One of the most remarkable features seen in the holotype is the presence of thin structures on both anterior sides of the specimen (Fig. 4a,b). They appear to surround the area where the stomach is assumed to be, which could imply that these thin structures could be gastric muscles.

The CT scan revealed another structure near the putative gastric muscles. Three pairs of lamellar structures are visible (Fig. 4c,d). They are thin, divided in leaf-like sections and resemble gills.

Additionally, a pair of elongated organs are located dorsally to the presumed gastric muscles and anteriorly to the supposed gills (Fig. 4e,f). There is a clear separation between the two parts of this tissue, thus suggesting that it is a paired structure.

### Remarks

Thylacocephalans are undoubtedly arthropods, but their affinities within this clade are still widely debated^1–3^. This lack of systematic resolution roots in the very incomplete knowledge of thylacocephalan anatomy. Therefore, exceptionally preserved materials such as those presented here can provide valuable anatomical details helping to better resolve trees comprising thylacocephalans. In the following, we discuss the structures that became visible in the CT-scan.

The cuticle displays a polygonal cuticular pattern separated by thin grooves, which are characteristic of thylacocephalans^9^. Secrétan^24^ and Broda *et al*.^9,25^ also suggested that the presence of a central depression in these polygons, the lumen (Fig. 3d), may represent pores where sensory setae inserted.

The eyes of *C. submarinus* have features strongly resembling the eyes of the Jurassic thylacocephalan *Dollocaris ingens* from La Voulte^26^, although the eyes of *Dollocaris* are significantly larger. Similarly, the Moroccan material has eyes composed of a dense and relatively regular pattern of hexagonal facets (Fig. 3b,c). However *C. submarinus* shows facets a little bit more elongate and a more hexagonal shape than *Dollocaris* in a similar surface area of the eye (Fig. 3e,f). The facets are also convex while those of *Dollocaris* appear to be more concave, probably due to post-mortem collapse^26^.

The thin and flattened, yet slightly inward curved structures (Fig. 4a,b) resemble muscles. The location on these structures (anterior and lateral) implies that there could have been an organ or other internal structure between. The position of the putative muscles surrounds the space where we would expect the stomach^3,26^. Thus, we interpret these structures as gastric muscles. Further remains of musculature are found on the anterior right side of the holotype. They lie above the area where the stomach likely used to be. We think that this could be remains of an anterior gastric muscle. Among the presumed gastric muscles (Fig. 4a,b), some can be tentatively interpreted as external mandible adductor muscles, similar to what has been described from decapod crustaceans^27^, but the possibility that these could be lateral gastric muscles cannot be ruled out until better preserved specimens reveal more anatomical details. No mandibles or other mouth parts have been reported from thylacocephalans so far, implying that the gastric muscle interpretation is more probable than being part of the mouth parts.

The leaf-like lamellar structures show three branches on each side (Fig. 4c,d). This could imply paired organs that are close to one another. The position of these structures is compatible with their interpretation as gills. However, gills normally should consist of eight pairs of lamellae, which is characteristic of thylacocephalans. Also, they are not as long as expected. This suggests that some lamellae are probably simply not preserved, just like most other internal organs and structures, or they were not visible in the CT-imagery due to an insufficient contrast in density.

The shape of the paired structure segmented in green (Fig. 4e,f) resembles that of gonads in some decapod crustaceans, which also concurs with the phylogenetic position of thylacocephalans. Kienbaum *et al*.^28^ discussed the gonads of decapods and explained that the ovaries are a pair of elongated organs located dorsally in the cephalothorax, a description fitting well with the structures found in the holotype. Although it is expected that gonads appear further posteriorly, a few species seem to have them more anteriorly than thought. Nagaraju^29^ detailed the same physiology and position in the body as Kienbaum *et al*.^28^, and located the ovaries on top of the stomach and hepatopancreas in some decapod crustaceans. The description of the hepatopancreas in crustaceans^30^ could fit the location of such structure, but this can be omitted here because thylacocephalans have a large hepatopancreas located ventrally in the thorax^26^. Although the gonad hypothesis stands a bit more than other interpretations like the hepatopancreas, further findings will contribute to revealing the correct interpretation of this structure.

*C. submarinus* differs from all concavicarids with its near mid-ventral fold. Species like *C. milesi* has a deep “U”-shaped optic notch^10^, which is not the case in *C. submarinus*. Also, the cuticle of *C. milesi* is terraced, unlike that of *C. submarinus*. *C. sinuata* has a deep carapace and a thick rostrum that extends to the anteroventral process^10^. This is not visible in *C. submarinus*. *C. submarinus* has a shorter carapace than *C. elytroides*^10,31^ and both have different types of cuticle; the cuticle of *C. elytroides* is striated while that of *C. submarinus* is much more similar to *C. bradleyi*. *C. submarinus* is a close relative of *C. bradleyi*^31^. They both have a convex carapace with a broad arch forming the dorsal margin. Both species have a similar outline with a rounded posterior end, a minute anteroventral process and a moderately-sized rostrum. Three differences distinguish these two species. First, the ventral margin of *C. submarinus* curves outwards while in *C. bradleyi*, the subcentral ventral margin is inflected inwards and upwards^31^. Secondly, *C. submarinus* shows a depression on the carapace reaching from near the curve to almost the rear end. Thirdly, the interior angle of the eye is sharper in *C. submarinus* than in *C. bradleyi*, here it is more rounded.

## Discussion

### Taphonomy and preservation

The Thylacocephalan Layer yields exceptionally preserved fossils of gnathostomes and thylacocephalans^20^. This includes poorly sclerotized structures such as the appendages of thylacocephalans and non-mineralized organs such as muscles and livers of chondrichthyans^20^. In line with the interpretations of Frey *et al*.^15,20^, we also assume that the exceptional preservation is linked with low oxygen levels of the bottom waters in the Maïder Basin. In the case of thylacocephalans, they demonstrated the presence of phosphate in the carapace, eyes and appendages, while the coarse crystals filling the void in the carapaces of most specimens are composed of yellow calcite. All thylacocephalans are embedded in flat red nodules; the red colour likely derives from haematite, which, in turn, probably goes back to weathered pyrite^20^.

Because their carapaces are thin or flexible, thylacocephalans often get deformed during diagenesis. The exceptionally well-preserved but slightly deformed material of *Concavicaris submarinus* corroborates that compaction of the sediments and the embedded fossils played a role (Fig. 1). Originally, the holotype of *C. submarinus* (PIMUZ 37349) was presumably broader when it was alive; parts of the legs are no longer attached to the body, the raptorial appendages are missing entirely, the eyes slightly shifted out of their position and one actually shifted towards the anteroventral process.

### Palaeoecology

Frey *et al*.^15^ reported the remarkable abundance of gnathostomes (mainly chondrichthyans) in the Thylacocephalan Layer, especially when compared to other regions and time periods where other non-vertebrates dominated. These thylacocephalans probably were important food sources for at least some of the Late Devonian chondrichthyans or large fishes as evidenced from, e.g., stomach contents in sharks^32,33^ and fish corpolites^34^. This is supported by previous studies that showed the co-occurrence of gnathostomes and crustaceans in the same layers in, e.g., Australia^32^ and the United States^33^ and by the association of thylacocephalans with remains of the shark *Cladoselache* from the Famennian Cleveland Shale^33^.

The invertebrate fauna from the Thylacocephalan Layer includes genus *Guerichia*^35^ (*elliptica* and *venusta*) and *Buchiola* (*Buchiola*)^36^, which are both bivalves known to have inhabited water bodies with a well-oxygenated upper water layer and low-oxygen conditions near the sea floor or they settled on the sediment in phases of better oxygenated bottom waters. Frey *et al*.^15^ highlighted the fluctuation of oxygen availability and its correlation with the global sea-level variation. This coincides with their hypothesis that oxygen levels decreased during this period due to reduced water exchange linked with a global regression^15^. Low oxygen conditions may have caused the sudden loss of numerous benthic and demersal species that require a higher level of oxygen to strive, while opportunists like *Guerichia* fared well. Thylacocephalans form the major part of the fauna during that time, thus indicating that these animals likely lived higher in the water column with normal oxygen content above the oxygen poor water.

**Figure 5:**
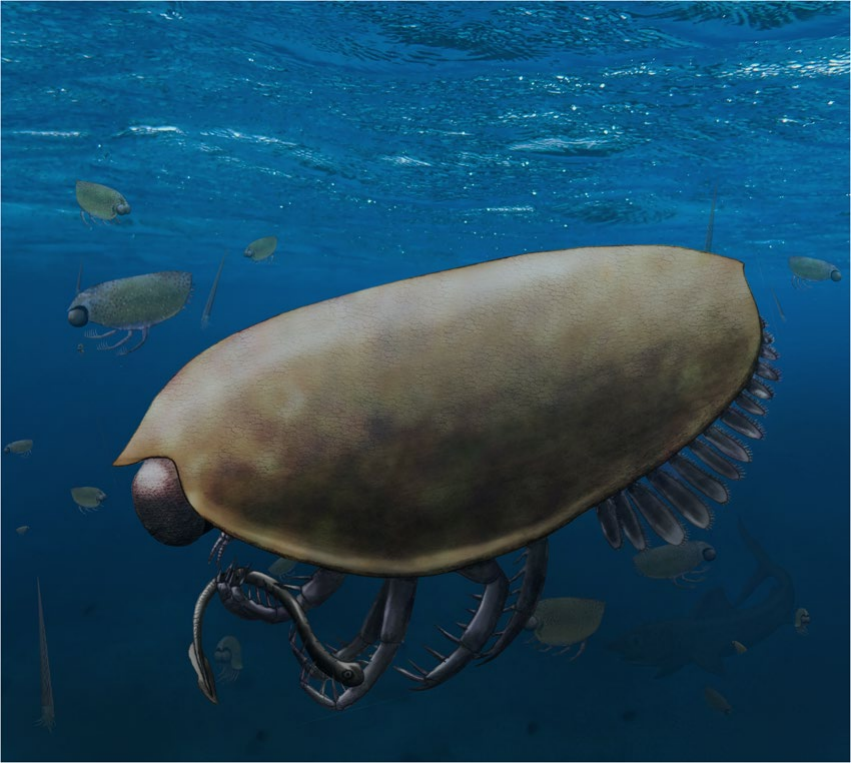
Reconstruction of *Concavicaris submarinus*, preying on a conodont animal, with a chondrichthyan in the background. Raptorial appendages are reconstructed with a size and shape intermediate between older (Silurian) and younger (Mesozoic) forms. Although direct evidence is missing that these thylacocephalans fed on conodonts, these are one of the few groups of which fossil remains are known from these strata. Predatory behavior of thylacocephalans was shown for younger relatives of *C. submarinus*.

Although the carapace of these thylacocephalans is slender and thus had a low drag, it enclosed most of the body including much of the appendages; this and the size of these paddle-like limbs do not suggest that they were capable of rapid swimming movements^26^. Their apposition eyes suggest an adaptation to normal light conditions in the euphotic zone^26^ rather than to dark environments as previously suggested^6^. Their round eyes facing in most directions including downwards corroborate a life in the water column. This is supported by the associated fauna and palaeoecological interpretations of other thylacocephalans^2,3^. Therefore, *Concavicaris submarinus* was most likely spending most of its life in the water column well above the low oxygen layers (Fig. 5). Since we did not find the raptorial appendages (possibly ripped off after death), we cannot infer much about their feeding behaviour. In accordance with findings from other localities and ages, it is the most parsimonious to assume that these animals did have large raptorial appendages and that they likely were ambush predators. Stomach contents are missing from the thylacocephalans of the Maider Basin, but their prey possibly consisted of smaller animals such as conodont animals, cephalopods etc.; a carnivorous mode of life has been demonstrated for at least some thylacocephalan species^6,26,37–39^. The missing raptorial appendages and absence of gut contents in these fossils makes it difficult to test these hypotheses yet, but future findings might help to test them.

## Conclusion

Here, we report the first thylacocephalans from Africa, namely from the middle Famennian of the Maider region of Morocco. We describe a new species of *Concavicaris*. The type material of *Concavicaris submarinus* is quite well preserved including fine details of the eyes, the cuticle, posterior appendages, putative gastric muscles and an undetermined paired organ. The presumed muscles indicate the location of the stomach in the species although the stomach itself is not preserved. Remarkably, the raptorial appendages are also not preserved but eight pairs of paddle-like appendages in the back could be documented. Based on comparisons with other thylacocephalan occurrences, we suggest that these Late Devonian thylacocephalans also were small predators and inhabited the middle to upper part of the water column. Due to their great abundance and co-occurrence with chondrichthyans and placoderms, we think that they represented an important food source for many fishes.

## Materials and Methods

This study is based on nine almost complete, well preserved, specimens (selected out of a much greater number in the field and in the lab). All specimens were collected in Morocco and are stored in the collections of the Palaeontological Institute and Museum of the University of Zurich, Switzerland (PIMUZ numbers) and at the Université Cadi Ayyad, Faculté des sciences et techniques, Département des sciences de la terre, Laboratoire Géosciences et Environnement in Marrakech (AA.MEM.DS.3 to 5).

Specimens were prepared using an air-scribe to remove the matrix of the iron-rich nodules. PIMUZ 37349 was CT-scanned with a Nikon XT H 225 ST tomography scanner at the University of Zurich (TIFF-stack as raw data with 1939 projections). The segmentation and 3D-model reconstruction was performed using the Mimics v.19 software (https://www.materialise.com/en/medical/software/mimics, Materialise, Leuven, Belgium).

The SEM images were obtained by imaging the surface of the specimen with a Jeol 6010 scanning electron microscope at the University of Zurich, in low vacuum conditions at 10 kV and 15 kV. Specimens were photographed with a Nikon D2x fitted with Nikon AF Nikkor 35–70 mm and AF Micro Nikkor 105 mm lenses.

## Data Availability

The original raw data file can be downloaded from Pangaea (10.1594/PANGAEA.913244).
